# Photoelectrochemical-Type Photodetectors Based on Ball Milling InSe for Underwater Optoelectronic Devices

**DOI:** 10.3390/nano15010003

**Published:** 2024-12-24

**Authors:** Yi Xu, Junxin Zhou, Dongyue Tian, Zhendong Fu, Yuewu Huang, Wei Feng

**Affiliations:** 1 College of Chemistry, Chemical Engineering and Resource Utilization, Northeast Forestry University, Harbin 150040, China; 18248700587@163.com (Y.X.); snowsmallface@163.com (J.Z.); tdy2873348364@nefu.edu.cn (D.T.); 2 Tianjin Jinhang Technical Physics Institute, Tianjin 300308, China; fzd119@outlook.com; 3 College of Materials Science and Chemical Engineering, Harbin University of Science and Technology, Harbin 150080, China; huangyuewu@hrbust.edu.cn

**Keywords:** Indium selenium, photoelectrochemical type, photodetector, self-powered, underwater optoelectronic devices

## Abstract

In this paper, InSe nanosheets were synthesized by a ball milling method, and photoelectrochemical-type photodetectors (PEC PDs) based on the ball milling InSe (M-InSe) were fabricated using simulated seawater as the electrolyte. M-InSe nanosheets show good absorption in the visible region of 450–600 nm. The M-InSe PEC PDs display a good self-powered photoresponse under 525 nm irradiation, including a high responsivity of 0.8 mA/W, fast response time of 28/300 ms, and good stability. Furthermore, the InSe PEC PDs successfully demonstrated prototype application in wireless underwater optical communication and optical imaging. These results demonstrate that M-InSe holds good application prospects in underwater optoelectronic devices.

## 1. Introduction

Underwater optoelectronic devices are foundational building blocks for ocean exploration, especially underwater optical communication (UOC), which has received substantial attention in recent years [1,2,3]. Some kinds of photodetectors (PDs) have been applied undersea to establish the UOC system, while complex packaging is indispensable [3,4,5]. The packaging process usually requires the use of organic polymers, which may cover the probe window and unavoidably weaken optical signals [5,6]. Photoelectrochemical-type (PEC) PDs are burgeoning devices, owning simple device structures without complex packaging and self-powered function [7,8,9]. Sun et al. first adopted artificial seawater as the electrolyte in PEC PDs based on p-AlGaN/n-GaN nanowires for portable and wireless UOC, demonstrating the potential applications of PEC PDs in marine environments [10]. However, the highest responsivity of p-AlGaN/n-GaN shows in the wavelength of 255 nm, which may not match well with the blue–green light transmitting window of seawater (450 nm to 550 nm) [2,6,10]. Therefore, it is attractive to develop high-performance PEC PDs with seawater-transmitting window coupled.

Indium selenide (InSe), an n-type 2D semiconductor with a bandgap of about 1.3 eV, has excellent room-temperature carrier mobility and adjustable bandgap with the thickness of nanosheets (NSs), which has been applied in advanced photoelectronic devices such as photodetectors, sensors, and field effect transistors [11,12,13]. Recently, InSe has been investigated for fabricating PEC PDs. Li et al. reported a liquid-phase exfoliated (LPE) method to prepare InSe NSs with a thickness of 7–8 atomic layer, showing stable PEC photoresponse in a KOH solution with a concentration of 0.2 M [14]. However, the responsivity is only 3.3 μA/W, and UV photoresponse in alkaline liquid environments limits their application in underwater optoelectronic devices. Yang et al. prepared InSe NSs with a thickness of 1.4–2.1 nm and a lateral size of 18 μm via an electrochemical exfoliation (ECE) method, the PEC PDs based on which shows a broadband photoresponse of 365–850 nm [15]. However, the ECE process was achieved based on an expensive and environmentally harmful intercalator of tetrabutylammonium bromide, limiting the large-scale application of InSe NSs. Ball milling, a mechanical exfoliation method, has been widely applied in the preparation of 2D materials due to its low cost, large amounts of preparation, and environmental friendliness [16,17]. Li et al. prepared graphene NSs from pristine graphite by a simple wet ball milling method. By adjusting the parameters of the ball milling method, the obtained graphene NSs show adjustable size, thickness, and electrochemical properties [18]. With the increase in the ball milling time from 4 h to 16 h, the size of the graphene NSs reduces gradually from 3.0 μm to 1.7 μm. The phenol-sensing system shows high sensitivity based on the ball milling graphene NSs, indicating the great potential of ball milling in semiconductor devices. Therefore, ball milling is a promising method for preparing InSe NSs in the preparation of PEC PDs.

Here, InSe NSs were synthesized using the glucose-assisted ball milling method. The crystallinity and morphology of ball milling InSe (M-InSe) NSs were determined by X-ray diffraction (XRD), transmission electron microscopy (TEM), atomic force microscopy (AFM), X-ray photoelectron spectroscopy (XPS), and UV-vis absorption spectrum. The M-InSe photoanodes were made using the drop-coating method on fluorine-doped tin oxide (FTO) glasses. The M-InSe PEC PDs achieved a high self-powered responsivity of 0.8 mA/W, fast response speed, and good stability under 525 nm irradiation in simulated seawater. The M-InSe PEC PDs were successfully applied in the wireless UOC system and underwater optical imaging, proving the application feasibility of M-InSe PEC PD in underwater optoelectronic devices.

## 2. Materials and Methods

Preparation of M-InSe NSs: First, the bulk InSe was grounded into powder. Then, 250 mg InSe powder, 2000 mg glucose, 670 mL DMF, and three different-sized ZrO_2_ balls (the diameters contenting of 3, 6, and 9 mm) were mixed in 100 mL ZrO_2_ jars. The jars were rotated into a planetary ball mill at 40 Hz for 4 h. The milled sample was ultrasonic processed in DI water for 30 min and wasted by DI water (3 times) and DMF (2 times). The ball-milled InSe NSs were extracted by the centrifuge (5 min at 500 rpm) and dried in a vacuum furnace at 353 K overnight. Finally, the InSe NSs were redispersed in DMF and reserved under vacuum conditions.

Characterizations of M-InSe NSs: The crystallographic phase of InSe (including bulk InSe and M-InSe NSs) was determined by X-ray diffraction (XRD, Rigaku Ultima IV, Tokyo, Japan). The microstructure of M-InSe NSs was observed by scanning electron microscopy (SEM, Thermo Apreo 2C, Waltham, MA, USA) and transmission electron microscopy (TEM, JEM-200, Tokyo, Japan). The elemental composition was measured by X-ray photoelectron spectroscopy (XPS, Thermo Scientific K-Alpha, Waltham, MA, USA). The optical absorption was characterized by the UV-vis absorption (Shimadzu UV-3600i Plus, Kyoto, Japan). The thickness of the M-InSe nanospheres was confirmed using atomic force microscopy (AFM, Nanoscope IIIa Vecco, Jersey City, NJ, USA).

Preparation of simulated seawater: Accurately weigh 6.684 g of NaCl, 0.050 g of NaHCO_3_, 0.873 g of Na_2_SO_4_, 0.181 g of KCl, 1.213 g of MgCl_2_·H_2_O, and 0.813 g of CaCl_2_ using an electronic balance. Add these substances to a beaker containing 250 mL of deionized water. Stir the mixture vigorously using a centrifuge stirrer for 30 min. After stirring, seal the beaker and store it in a dark place for later use.

Fabrication and PEC performance of M-InSe PEC PDs: The M-InSe NSs were applied on the precleaned FTO substrates using the drop-coating method (effective area of 1 × 1 cm^2^) and dried for 6 h in a vacuum desiccator with a set temperature of 373 K. Then, the M-InSe photoanodes were annealed under an argon atmosphere at 623 K for 40 min. The PEC PD is a simplified two-electrode system including a working electrode of M-InSe photoanodes and a counter electrode of Pt wire using simulated seawater as the working electrolyte. The PEC photoresponse properties were tested using an electrochemical workstation (CHI660E, CHENHUA, Shanghai, China) and an LED light source (PLSLED100C, including the LED lights and controller) with different wavelengths at power densities. The UOC system is measured by a pocket electrochemical analyzer device (CS100, CORRTEST, Wuhan, China) with a two-electrode system.

## 3. Results and Discussion

**Scheme 1:**
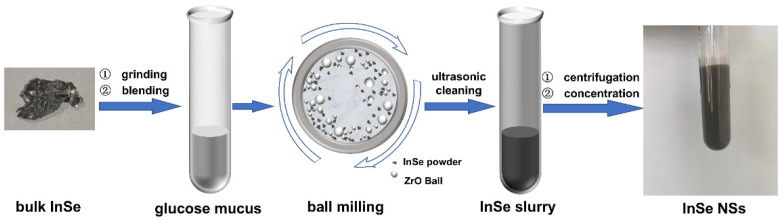
The schematic preparation process of M-InSe NSs by ball milling.

**Figure 1:**
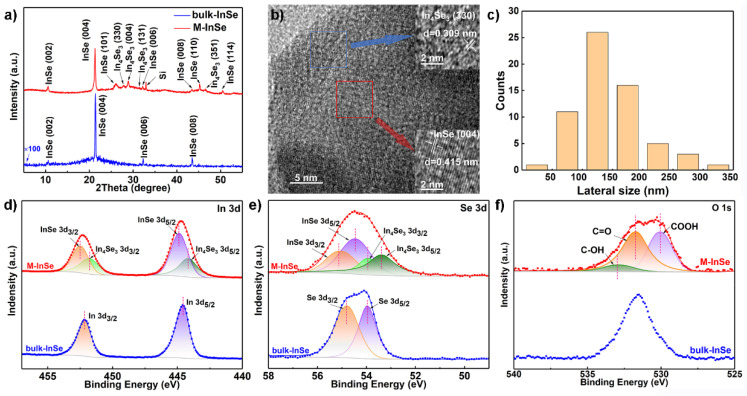
Characterization of M-InSe. (**a**) XRD patterns of M-InSe NSs and bulk InSe. (**b**) HRTEM image of M-InSe. (**c**) The lateral size distribution of M-InSe NSs. (**d**–**f**) XPS spectra of In 3d, Se 3d, and O 1s.

Scheme 1 briefly shows the ball milling process of InSe NSs from bulk InSe (more details are provided in Section 2) and the obtained InSe NSs are named as M-InSe. Figure 1a presents the XRD patterns of bulk InSe and M-InSe NSs. The main XRD peaks match well with the original bulk InSe (PDF 34-1431) [19], and other weak peaks confirm that a small amount of In_4_Se_3_ (PDF 51-0808) [20] is present in the M-InSe samples. The ball milling generates a higher temperature and continuously machining the InSe sample, which inevitably brings some Se vacancy (V_Se_) defects in the InSe, finally converting to In_4_Se_3_ [21,22,23]. The high-resolution transmission electron microscopy (HRTEM) and Fourier transform patterns of M-InSe NSs in Figure 1b further confirm the existence of InSe and In_4_Se_3_. The red area that zoomed in the inset of Figure 1b shows a lattice spacing of 0.415 nm, which is consistent with the (004) lattice plane of InSe [24], while the blue area displays a lattice spacing of 0.309 nm which is indexed to the (330) lattice plane of In_4_Se_3_ [25]. As shown in the SEM images (Figure S1), the drop-coated M-InSe NSs are evenly distributed on the FTO substrate, forming a uniform thin film and showing typical NS morphology. TEM was conducted to characterize the morphology of M-InSe further, as shown in Figure S2. M-InSe represents layered morphology, and the lateral sizes are mainly distributed in 50–200 nm (Figure 1c). Different from bulk InSe, each XPS In 3d spectra of M-InSe can be divided into two peaks, as shown in Figure 1d. The peaks at 444.9 and 452.5 eV belong to In 3d_5/2_ and In 3d_3/2_ of InSe [24,26], and the peaks at 444.2 and 451.8 eV correspond to In 3d_5/2_ and In 3d_3/2_ of In_4_Se_3_ [25]. The Se 3d spectra of M-InSe also contain InSe and In_4_Se_3_ (Figure 1e). Notably, the peaks including In 3d and Se 3d of In_4_Se_3_ shift to the lower binding energy side compared with the bulk InSe, which can be explained by the residual electrons of V_Se_ flowing to the In and Se atoms, reducing their bending energy after obtaining electrons, and sheltering the interaction between their charged nucleus and inner shell electrons [27,28]. The XPS results further demonstrate that the M-InSe is composed of InSe and In_4_Se_3_. Compared with bulk InSe, the In 3d and Se 3d XPS peaks of the InSe in the M-InSe samples take redshift, which is attributed to oxygen doping in ball milling processes, confirmed by the O 1s XPS peaks (Figure 1f) [15,26].

**Figure 2:**
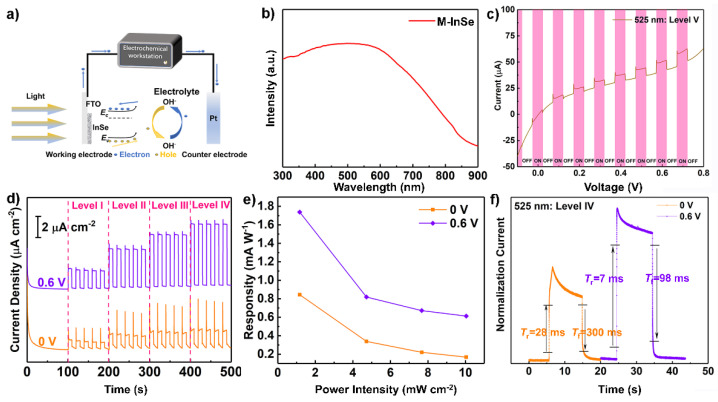
Photoresponse of the M-InSe PEC PDs using simulated seawater as the electrolyte: (**a**) The schematic diagram of the working mechanism of the M-InSe PEC PDs. (**b**) UV-vis-NIR absorption spectrum of M-InSe. (**c**) The LSV curve of the M-InSe PEC PDs. (**d**) The *J*-t curves irradiated by 525 nm with different light power densities. (**e**) The responsivity. (**f**) The response speed.

M-InSe NSs photoelectrodes were fabricated using the drop-coating method to investigate their PEC photoresponse behaviors (more details can be found in the Section 2). The schematic diagram of the M-InSe PEC PDs with a two-electrode system is shown in Figure 2a, consisting of M-InSe photoanodes (1 × 1 cm^2^) as the working electrode, a counter electrode (Pt wire), and electrolyte (simulated seawater). The typical optical images of M-InSe photoanodes are shown in Figure S3. Due to a built-in electric field at the interface of M-InSe and electrolyte, M-InSe PEC PDs own self-driven function [14,15] (more details in the Supplementary Materials). The working mechanism of the M-InSe PEC PDs is shown in Figure 2a. As an n-type semiconductor, the majority carrier of InSe is the free electron, and when InSe contacts with the electrolyte, the electrons start free diffusion to the surface of the electrolyte, forming the built-in electric field pointing from the M-InSe to the electrolyte. When the light with the corresponding wavelength shines on the M-InSe, the photogenerated electrons and holes are separated by the built-in electric field. Then, the photogenerated electrons enter the external circuit through the conducting substrate and reduce OH· on the surface of the counter electrode (reaction Equation (1)). The photogenerated holes oxide the OH− at the InSe/electrolyte interface (reaction Equation (2)).

OH− + h+ → OH,(1)

OH· + e− → OH−,(2)

Thus, once a cycle of the PEC process is completed, the photocurrent is formed.

As shown in Figure 2b, the M-InSe samples show good absorption in the visible light region of 450–600 nm, which matches well with the absorption blind area of the seawater (450 nm to 550 nm) [2,29]. As shown in Figures S4 and S5, the thickness of the M-InSe NSs sample is mainly below 30 nm, offering a bandgap of 1.36 eV for M-InSe NSs. Therefore, the M-InSe PEC PDs were irradiated by 525 nm light in this study. Figure 2c shows the linear sweep voltage curve (LSV) of the M-InSe PEC PDs irradiated by the pulse 525 nm light. M-InSe PEC PDs display self-powered function and the photoresponse gradually enhances with the increase in bias voltage. The photocurrent density (*J*_ph_), the difference between current density (*J*) with and without irradiation, is 0.98 and 2.03 μA/cm^2^ for 0 and 0.6 V, respectively. Figure 2d shows a distinct transient photoresponse in self-powered mode, which is attributed to photogenerated carrier recombination induced by surface defects [30]. By applying a bias voltage of 0.6 V, the transient photocurrent was suppressed due to accelerating the photogenerated carrier separation and transport under external bias potential driven. To further evaluate the photoresponse behavior of InSe PEC PDs, the responsivity (*R*) and specific detectivity (D*) values were calculated by the following equations of *R* = *J*_ph_/*P* and D* = *R* × S^0.5^/(2 × *S* × *q* × *J*_d_)^0.5^, respectively, where *P*, *S*, *q*, and *J*_d_ represent relevant light power intensity, the area of effective exposure area (1 × 1 cm^2^), the charge of an electron (1.6 × 10^−19^ C), and the dark current density, respectively. At the light power density of level I, the *R* is 2.03 and 0.98 mA/W for 0.6 and 0 V, respectively. Meanwhile, the corresponding D* is 2.97 × 10^9^ Jones and 3.19 × 10^9^ Jones (more data are listed in Tables S1–S4 of Supplementary Materials). The *R*-value exceeds some reported InSe-based PEC PDs, such as 0.0078 mA/W of InSe (Ge)/InSe [31] and 0.0033 mA/W of liquid-exfoliated InSe [14], showing the better PEC photoresponse via the ball milling prepared InSe NSs. However, compared to the 7.21 mA/W of the pure InSe NSs (525 nm irradiation) [15], the photoresponse of M-InSe declines, which may be due to the adverse effects of In_4_Se_3_. The crystal damage caused by excessive V_se_ such as crack formation results in the degradation of the PEC performance [32].

The response time is another indispensable parameter to evaluate the information transmission capability of PEC PDs. As shown in Figure 2f, the response time is 28 and 300 ms for the rise process (the time from 10% to 90% of *J*_ph_) and decay process (the time from 90% to 10% of *J*_ph_) for the self-powered M-InSe PEC PDs, respectively. The response times are reduced to 7 and 98 ms at 0.6 V, respectively, due to the accelerated carrier transport and interfacial charge transfer under external bias [33,34,35]. Previous reports have demonstrated that InSe NSs can be oxidized in air [36]. After being exposed to air for four weeks, the characteristic peaks in the Raman spectrum of InSe NS with a thickness of 6 nm disappeared. Thus, the M-InSe was placed in a vacuum storage tank for preservation. In that case, the M-InSe PEC PDs displayed excellent long-term stability and the photoresponse was 80% of the original value after ten months of storage, as shown in Figure S6, further indicating the good potential in practical application.

**Figure 3:**
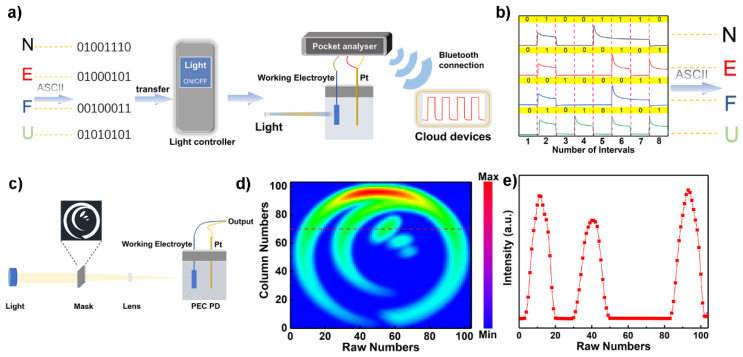
The demonstration of prototype applications. (**a**) Schematic diagram of the wireless UOC based on the M-InSe PEC PDs. (**b**) The input process of information transcoding corresponding to the ASCII codes of the four letters “NEFU” and the corresponding waveforms of the output data. (**c**) Schematic diagram of the imaging system based on the M-InSe PEC PDs. (**d**) Image obtained from the image scanning system at a bias of 0 V. (**e**) Signal detection along the red line in (**d**).

Finally, a wireless optical communication system of UOC was set up, consisting of the M-InSe PEC PD, a mobile phone, a pocket electrochemical analyzer, LED lights, and a light source controller. The optical image of the UOC system is shown in Figure S7. Figure 3a describes the process of information transcoding input to the light controller. Here, the input data (“NEFU”) was first converted into four binary strings via the ASCII code [37]. The modulated LED light can switch regularly according to the input of the strings (“1” and “0” can be represented by the light on and off) controlled by the controller. The modulated optical signals are received by the M-InSe PEC PDs via the electrochemical analyzer, which are converted to electrical signals, and then transmitted to portable devices (mobile phones) via Bluetooth. By analyzing and decoding the electrical signals, the transmitted data of four letters “NEFU” was obtained. Figure 3b illustrates a comparison between the received signals and the original strings. Notably, the photocurrent signals were identical to the original input data. Therefore, we have successfully demonstrated wireless data communication without a complex packaging process using simulated seawater.

Further, the M-InSe PEC PDs are employed in an imaging system to confirm their capacity for underwater image applications, and the corresponding schematic diagram of the imaging system and process are shown in Figure 3c. The inset of Figure 3c displays the original image of the mask plate. Figure 3d shows a 107 × 103-pixel image measured with a bias potential of 0 V in simulated seawater. The obtained image is close to the same as the complex initial image of the mask version. Figure 3e shows the normalization signals of the red line in Figure 3d, and the rapid increases and decreases in signal intensity prove the reliability when applied to underwater imaging. The above results indicate that the M-InSe PEC PDs have great application prospects in underwater optoelectronic devices.

## 4. Conclusions

In summary, we successfully synthesized M-InSe NSs by the ball milling method and explored the photoresponse and application prospects of M-InSe-based PEC PDs using simulated seawater as an electrolyte. The InSe PEC PDs show good self-powered capability under 525 nm light irradiation with a high responsivity of 0.8 mA/W, a fast response speed of 28/300 ms, and good stability. The UOC and optical imaging systems were used to demonstrate the application potential of self-powered M-InSe PEC PDs in underwater optoelectronic devices.

## Data Availability

The original contributions presented in this study are included in the article/supplementary material. Further inquiries can be directed to the corresponding author.

## Supplementary Materials

The following supporting information can be downloaded at https://www.mdpi.com/article/10.3390/nano15010003/s1. The SEM, TEM, AFM, Tauc curve, and photograph of M-InSe; the working mechanism of the PEC PDs; the long-term stability; the optical images of the UOC system; the power density of light source; and calculated *R*, *D**, and *J*_ph_.
