# Supplementary material for: Photoelectrochemical-Type Photodetectors Based on Ball Milling InSe for Underwater Optoelectronic Devices
Source: Nanomaterials (Basel). 2024 Dec 24;15(1):3. doi: 10.3390/nano15010003 (PMC11723190; doi:10.3390/nano15010003)
Supplement: Supplementary file 1 [file nanomaterials-15-00003-s001.zip › nanomaterials-3391907-Supplementary.pdf]

Photoelectrochemical-Type Photodetectors Based on Ball-Milling InSe for Underwater Optoelectronic Devices

Yi Xu [1,#], Junxin Zhou [1,#], Dongyue Tian [1], Zhendong Fu [2], Yuewu Huang [3] and Wei Feng [1,*]

1 College of Chemistry, Chemical Engineering and Resource Utilization, Northeast Forestry University, Harbin, 150040, China; 18248700587@163.com (Y.X.); snowsmallface@163.com (J.Z.); tdy2873348364@nefu.edu.cn (D.T.)

2 Tianjin Jinhang Technical Physics Institute, Tianjin, 300308, China; fzd119@outlook.com (Z.F.)

3 College of Materials Science and Chemical Engineering, Harbin University of Science and Technology, Harbin, 150080, China; huangyuewu@hrbust.edu.cn (Y.W.)

* Correspondence: wfeng@nefu.edu.cn

Email: wfeng@nefu.edu.cn

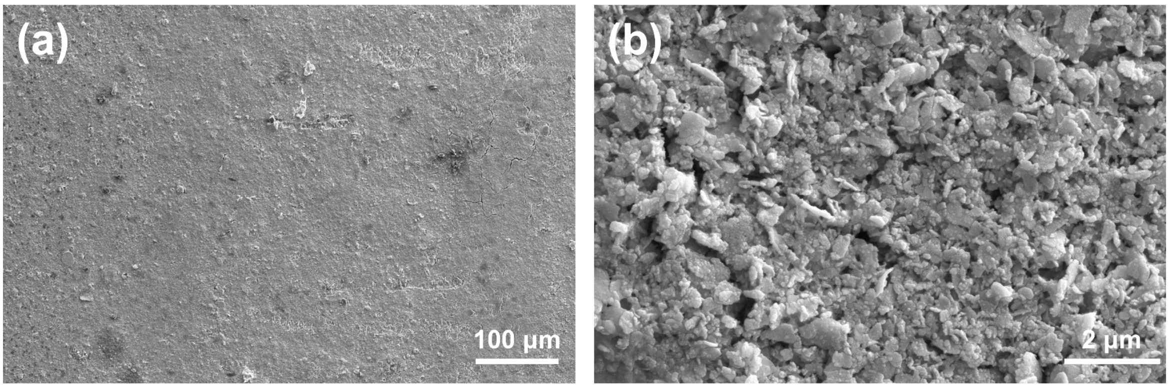

Figure S1. SEM images of M-InSe NSs.

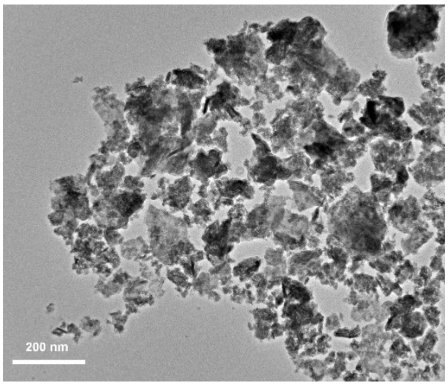

Figure S2. TEM image of M-InSe NSs.

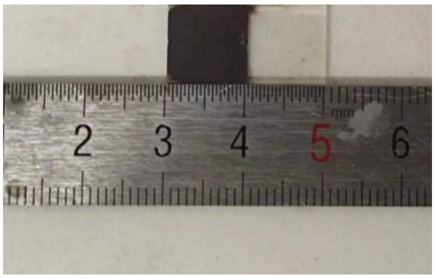

Figure S3. The photograph of M-InSe photoanodes.

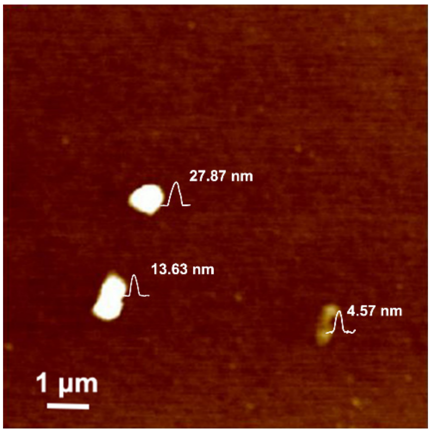

Figure S4. AFM picture of M-InSe NSs.

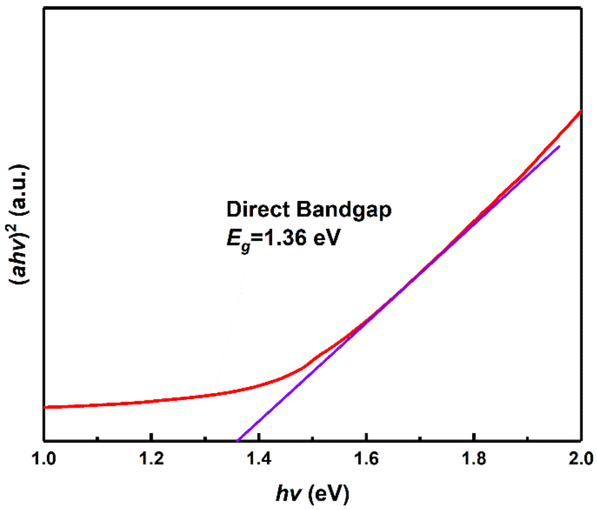

Figure S5. Tauc curve of M-InSe NSs.

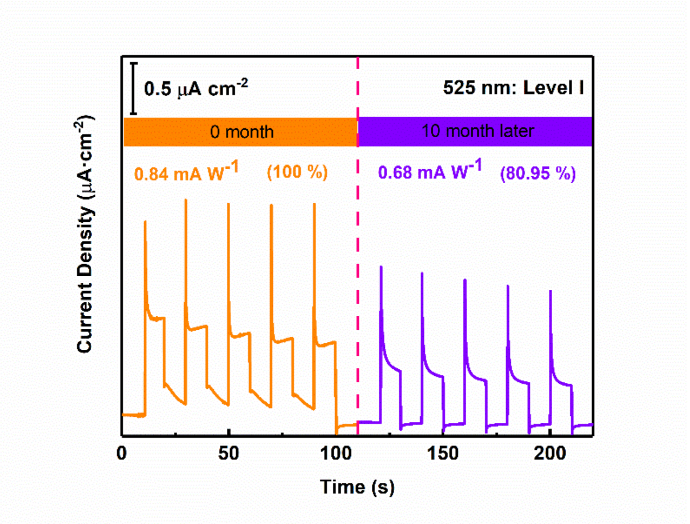

Figure S6. The contract of fresh and 10 months storage of M-InSe PEC PDs.

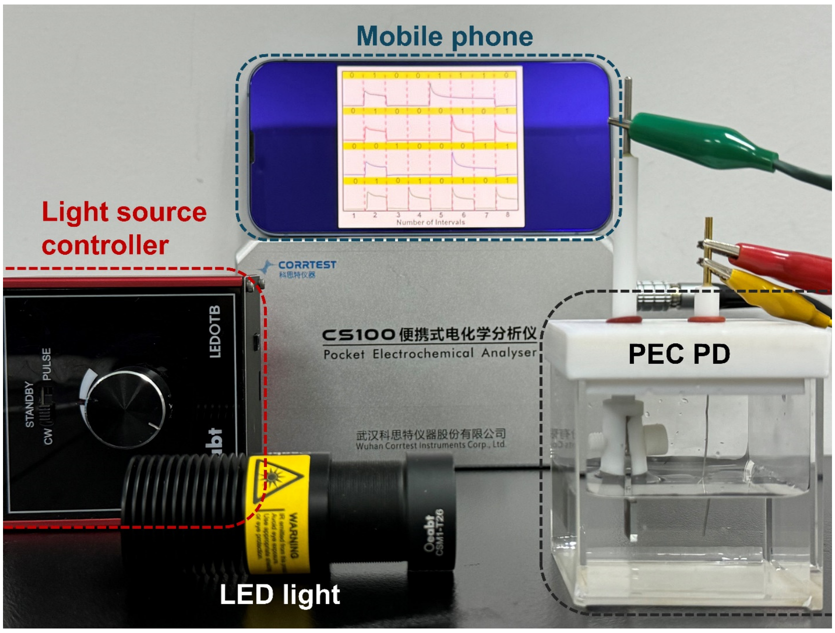

Figure S7. The optical image of the UOC system, where the switch of LED is controlled by modulation waveform.

Table S1. Light power intensity of 525 nm light sources.

P (mW/cm^2) / Light (nm)	Level I	Level II	Level III	Level IV
525	1.17	4.73	7.67	10.04

Table S2. J_{ph} of M-InSe PEC PDs for 525 nm wavelength of light with different power intensities at external bias potential of 0 V and 0.6 V in simulated sea water.

I_{ph} (μA/cm^2) / Light (nm)	Level I	Level II	Level III	Level IV
525 (0 V)	0.98	1.60	1.69	1.70
525 (0.6 V)	2.03	3.87	5.15	6.15

Table S3. R values of M-InSe PEC PDs for 525 nm wavelength of light with different power intensities at external bias potential of 0 V and 0.6 V in simulated sea water.

R (mA/W) / Light (nm)	Level I	Level II	Level III	Level IV
525 (0 V)	0.84	0.34	0.22	0.17
525 (0.6 V)	1.73	0.81	0.67	0.61

Table S4. D* values of M-InSe PEC PDs for 525 nm wavelengths of light with different power intensities at external bias potential of 0 V and 0.6 V in simulated seawater.

D^* (10^9 Jones) / Light (nm)	Level I	Level II	Level III	Level IV
525 (0 V)	3.19	1.28	0.83	0.64
525 (0.6 V)	2.97	1.40	1.15	1.05
